# Promotion of Reprogramming to Ground State Pluripotency by Signal Inhibition

**DOI:** 10.1371/journal.pbio.0060253

**Published:** 2008-10-21

**Authors:** Jose Silva, Ornella Barrandon, Jennifer Nichols, Jitsutaro Kawaguchi, Thorold W Theunissen, Austin Smith

**Affiliations:** 1 Wellcome Trust Centre for Stem Cell Research, University of Cambridge, Cambridge, United Kingdom; 2 Department of Biochemistry, University of Cambridge, Cambridge, United Kingdom; 3 Department of Physiology, Development, and Neuroscience, University of Cambridge, Cambridge, United Kingdom; Baylor College of Medicine, United States of America

## Abstract

Induced pluripotent stem (iPS) cells are generated from somatic cells by genetic manipulation. Reprogramming entails multiple transgene integrations and occurs apparently stochastically in rare cells over many days. Tissue stem cells may be subject to less-stringent epigenetic restrictions than other cells and might therefore be more amenable to deprogramming. We report that brain-derived neural stem (NS) cells acquire undifferentiated morphology rapidly and at high frequency after a single round of transduction with reprogramming factors. However, critical attributes of true pluripotency—including stable expression of endogenous Oct4 and Nanog, epigenetic erasure of X chromosome silencing in female cells, and ability to colonise chimaeras—were not attained. We therefore applied molecularly defined conditions for the derivation and propagation of authentic pluripotent stem cells from embryos. We combined dual inhibition (2i) of mitogen-activated protein kinase signalling and glycogen synthase kinase-3 (GSK3) with the self-renewal cytokine leukaemia inhibitory factor (LIF). The 2i/LIF condition induced stable up-regulation of Oct4 and Nanog, reactivation of the X chromosome, transgene silencing, and competence for somatic and germline chimaerism. Using 2i /LIF, NS cell reprogramming required only 1–2 integrations of each transgene. Furthermore, transduction with Sox2 and c-Myc is dispensable, and Oct4 and Klf4 are sufficient to convert NS cells into chimaera-forming iPS cells. These findings demonstrate that somatic cell state influences requirements for reprogramming and delineate two phases in the process. The ability to capture pre-pluripotent cells that can advance to ground state pluripotency simply and with high efficiency opens a door to molecular dissection of this remarkable phenomenon.

## Author Summary

Development of an organism proceeds irreversibly from embryo to adult, with cells differentiating progressively towards specialised final phenotypes. Now, following the pioneering discovery of induced pluripotency by Shinya Yamanaka, it has become possible to reverse developmental time: we can reprogramme an adult cell back to the naïve state of pluripotency found in the early embryo. Induction of pluripotency is an extraordinary phenomenon but is currently poorly understood and inefficient. We investigated stem cells from the mouse brain and found that they reprogrammed faster than other cell types. However, the reprogrammed brain cells arrested on the verge of full pluripotency and did not gain some essential properties of induced pluripotency. Guided by the rationale of reversing a development process, we explored the effect of blocking the signal that initiates loss of pluripotency and entry into differentiation in the embryo. We used a chemical inhibitor of this signal in combination with stimulation of a second pathway known to promote maintenance of pluripotency. This simple treatment allowed the partly converted neural stem cells to complete the transition efficiently and become indistinguishable from embryonic stem cells. Therefore, incompletely reprogrammed cells, which have previously been dismissed as useless by-products of attempts to generate pluripotent stem cells, in fact provide the fastest, most reliable, and most efficient route to obtaining authentic induced pluripotent cells.

## Abstract 3

Induced reprogramming of stem cells proceeds in two phases via an intermediate that is undifferentiated but not pluripotent. Inhibition of mitogen-activated protein kinase signaling converts this intermediate transitional state to authentic pluripotency.

## Introduction

The process by which a fraction of somatic cells are converted into a quasi or fully pluripotent state following transfection with reprogramming genes [1,2] is obscure and difficult to elucidate with current methodologies. The differentiation state of a somatic cell may be a critical parameter determining the requirements for, the efficiency of, and the kinetics of induced pluripotent stem (iPS) cell generation. Studies to date have used heterogeneous starting populations for the production of iPS cells [2–4] or have involved a prior genetic manipulation to dedifferentiate mature cells [5]. Consequently, the identity of those target cells that actually become reprogrammed is uncertain. Mouse brain–derived neural stem (NS) cells can be stably propagated as undifferentiated clonal populations in adherent serum-free culture [6–8] (Figure S1A). These cells have central nervous system–restricted potency to differentiate into neurons, astrocytes, and oligodendrocytes. However, in adherent culture in epidermal growth factor (EGF) plus fibroblast growth factor (FGF) the level of neuronal or glial differentiation is less than 1% [6,8]. Previous studies have shown that NS cells can be reprogrammed efficiently by either embryonic stem (ES) cell fusion or oocyte nuclear transfer [9,10]. We asked whether NS cells derived from foetal and adult brain can be efficiently converted into pluripotent cells by gene transduction and whether the reprogramming process can be promoted using defined conditions for optimal propagation of ground state pluripotent stem cells derived from the mouse blastocyst [11].

## Results

### NS Cells Undergo Rapid and Efficient Conversion towards a Pluripotent State

**Figure 1:**
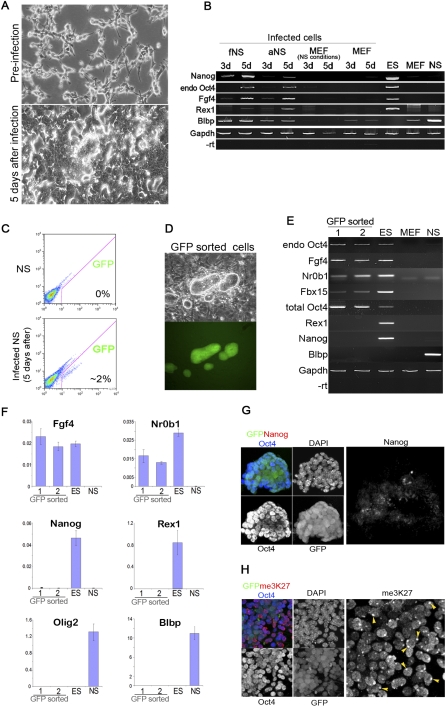
NS cells Undergo Rapid but Incomplete Conversion Towards a Pluripotent Phenotype (A) Phase contrast images of NS cells in standard culture (upper) and 5 d after infection with the four factors (lower). (B) RT-PCR analyses for Nanog, endogenous (endo) Oct4, Fgf4, Rex1, and Blbp in infected foetal (fNS) and adult (aNS) cells 3 and 5 d d after infection. MEF infections under both MEF and NS cell culture conditions were analysed in parallel. Only at later time points, after 10 d, did a few colonies of ES cell-like morphology emerge from infected MEFs. (C) Flow cytometry analysis of Oct4-GFP activation in NS cells 5 d after infection. (D) Phase and fluorescence images of FACS purified GFP-expressing cells cultured on feeders. (E and F) Conventional (E) and quantitative (F) RT-PCR analyses of Oct4GFP expressing cells expanded on feeders. (G) Immunofluorescence staining for Oct4 and Nanog. (H) Staining for me3K27. Arrowheads indicate the nuclear body diagnostic of the inactive X chromosome.

We first infected mouse embryo fibroblasts (MEFs) with retroviruses harbouring the reprogramming factors Oct4, Sox2, c-MycT58, and Klf4 exactly as described [2,12]. Individual colonies of undifferentiated morphology expressing an Oct4-enhanced green fluorescent protein (GFP) reporter transgene [10,13] emerged after 3 wk, similar to previously reports [14]. The frequency of NS cells infected with a GFP reporter retrovirus (70%–80%) is similar to that observed with MEFs (Figure S1B), suggesting that NS cells should be infected with reprogramming vectors at similar efficiency. We therefore infected NS cells with the same retroviruses used to reprogramme MEFs. In striking contrast to MEFs, a high proportion of infected NS cells acquired a similar appearance to undifferentiated embryonic stem (ES) cells only 3 d after infection. Two d later, plates were confluent with ES cell–like cells and in need of passaging (Figure 1A). Concomitant with the morphological conversion, both foetal and adult NS cell–derived cultures exhibited expression of ES cell markers Fgf4, Rex1, Nanog, and endogenous Oct4 at the transcript level (Figure 1B), while these remained undetectable in parallel MEF infections at these time points. However, levels appeared lower than in ES cells, and only occasional cells showed credible immunostaining for Nanog protein (Figure S1C).

In female cells, loss of silencing marks on the X chromosome is a diagnostic feature of full pluripotent status and provides unambiguous demonstration of major epigenetic erasure [10,15]. Immunostaining against the silent chromatin mark trimethyl H3-K27 (me3H3K27) readily identifies the inactive X chromosome [16]. The nuclear body of intense staining corresponding to a persisting inactive X chromosome was evident in all nuclei of infected XX cells (Figure S1D). Therefore, despite acquiring some similarities to ES cells, these cells have not attained true pluripotent identity.

### NS Cell Reprogramming Stalls at an Intermediate State

To investigate whether further reprogramming might occur in a small proportion of cells, we examined the expression of the Oct4-GFP transgene that has been shown to reactivate when the differentiated genome acquires pluripotency [10,13]. In infected MEFs, we did not detect Oct4 reporter activity until 3 wk after infection, as previously reported [14]. For NS cells, GFP was evident 5 d after infection, but in only 2% of cells (Figure 1C). We purified GFP-positive cells by flow cytometry and expanded them on a feeder layer (Figure 1D). In this population, both Fgf4 and endogenous Oct4 mRNA are detectable at similar levels as in ES cells (Figure 1E and 1F). Fbx15 and NrOb1 transcripts are also present, although at lower levels. However, Nanog and Rex1 mRNAs are minimal and Nanog protein is undetectable (Figure 1F and 1G). In contrast, total Oct4 mRNA levels are elevated compared to ES cells, indicating failure to silence the retroviral transgene (see also Figure S3A). Consistent with incomplete epigenetic reprogramming, the inactive X chromosome persists uniformly in these cells (Figure 1H). Strikingly, Oct4 reporter expression was unstable and lost entirely within two passages. A population expressing GFP could only be obtained by repeated fluorescence-activated cell sorting (FACS) purification at each passage. Blastocyst injection of GFP-positive cells did not yield chimaeras, confirming that full pluripotent status is not attained. These observations indicate that neither morphology nor Oct4 reporter activation are reliable surrogates for designation of pluripotent identity. Fallibility of the Oct4 reporter is not, in fact, surprising given that Oct4 is broadly expressed throughout the pre-pluripotent phase of embryo development and subsequently in the post-implantation egg cylinder [17,18] and derivative epithelialised epiblast stem cells (EpiSCs) [19,20]. Furthermore our findings are consistent with the original description of incomplete induced reprogramming by Takahashi and Yamanaka [2] using selection for Fbx15 and the subsequent report that even following selection for expression of a Nanog transgene, 95% of colonies do not achieve stable activation of the endogenous gene and authentic pluripotency [1]. Indeed we found that infections of MEFs yielded colonies of ES cell–like morphology that could readily be expanded but remained negative for expression of endogenous pluripotency genes and did not stably activate the Oct4-GFP reporter. Interestingly, these colonies emerged at around 14 d, while rarer colonies that did express Oct4-GFP only appeared from 3 wk onwards.

### Modulation of Intracellular Signaling Allows Progression to Authentic Pluripotency

**Figure 2:**
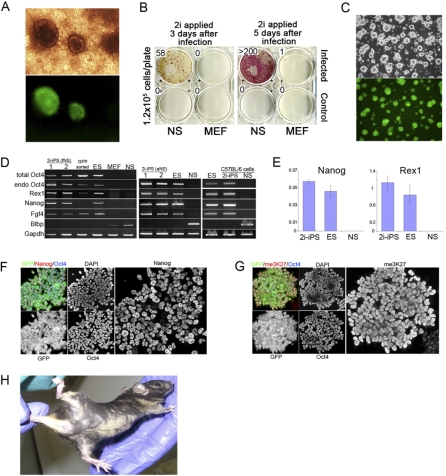
MEK and GSK3 Inhibitors (2i) Promote Reprogramming to Full Pluripotency (A) Example of 2i-iPS cell colonies generated from aNS cells replated at day 5 after infection in 2i medium. (B) Plates containing control and infected aNS cells and MEFs were cultured in 2i media from day 3 or day 5 after infection and stained for AP 10 d later. Numbers of AP-stained colonies are indicated. (C) Established 2i-iPS cell line. (D and E) Conventional (D) and quantitative (E) RT-PCR analyses of 2i-iPS cells generated from aNS and fNS Oct4GFP NS cells, and from fNS cells of non-transgenic C57BL/6 strain background. (F) Immunofluorescence staining for Oct4 and Nanog in 2i-iPS cell colony after first passage. (G) Immunostaining forme3K27. A cluster of residual Oct4-negative cells retains the nuclear body of an inactive X chromosome, providing an internal control. (H) Chimera generated from injection of 2i-iPS (aNS) cells into C57BL/6 host blastocyst.

We then investigated whether the partially reprogrammed cells could attain full pluripotent status. We applied molecularly defined conditions designed to sustain ES cells in a pluripotent ground state by neutralizing inductive differentiation stimuli [11,21]. This comprises serum-free culture in the presence of two small molecule inhibitors (2i) of the Mek (mitogen activated protein kinase)/Erk (extracellular signal regulated kinases 1 and 2) pathway and of GSK3, respectively. We also included the cytokine LIF, which maximizes clonogenic self-renewal of ES cells [11,22,23]. An additional feature of these culture conditions is that they do not support propagation of most somatic cell types nor of EpiSCs, which are generally reliant on Erk activity for growth and proliferation. Infected, morphologically converted cells were trypsinized and replated at day 5 into serum-free 2i/LIF medium. Multiple colonies grew up, two-thirds of which expressed the Oct4-GFP reporter in all undifferentiated cells (Figure 2A). We then investigated application of serum-free 2i/LIF directly to the infected population after 3 or 5 d. Noninfected control cells died or ceased proliferation. At most, one or two colonies per plate were obtained from infected MEFs when 2i was applied at these early time points. In contrast, abundant alkaline phosphatase–positive undifferentiated colonies emerged on the NS cell plates, more when 2i was applied at day 5 than at day 3 (Figure 2B). Many colonies (60%) showed activation of the Oct4 reporter transgene. Passaged plates or individually picked colonies yielded cell lines with ES cell morphology that could be expanded continuously in the absence of selection without loss of Oct4 reporter activity (Figure 2C). Similar results were obtained from foetal NS cells including from a wild-type strain C57BL/6-derived line without recourse to any transgene reporter. MEFs, by contrast, yielded few colonies per plate and only consistently when inhibitors were applied more than 2 wk after infection (Figure S2). In addition, we noted a marked effect of strain background, such that 20-fold more colonies were obtained from strain 129 MEFs compared with the mixed background Oct4-GFP MEFs.

Regardless of origin, 2i-iPS cells express Nanog, Rex1, and Oct4 mRNAs at levels similar to ES cells (Figure 2D and 2E). Total Oct4 mRNA is comparable to that observed in ES cells. The indication that the retroviral transgene has become silenced is confirmed by Northern hybridisation (Figure S3A). Immunofluorescent staining revealed expression of nuclear localised Nanog (Figure 2F). Erasure of epigenetic silencing marks on the X chromosome is evidenced by loss of the me3H3K27 nuclear body (Figure 2G) and by expression of Tsix from both X chromosomes (Figure S3B).

2i-iPS cells could be maintained upon transfer from 2i/LIF into medium containing either serum or Bone Morphogenetic Protein (BMP) in the presence of LIF. They differentiated in the absence of LIF without 2i (Figure S3C and S3D), mimicking ES cell dependency [22–24]. The definitive demonstration of ground state pluripotency is colonization of viable chimaeric mice. We therefore injected 2i-iPS cells into blastocysts. Liveborn chimeras were obtained that have developed into healthy adulthood (Figure 2H). This establishes that the reprogrammed cells can respond fully to instructive cues in the developing embryo and have authentic pluripotency.

### Ground State Pluripotency Is Induced by 2i/LIF

**Figure 3:**
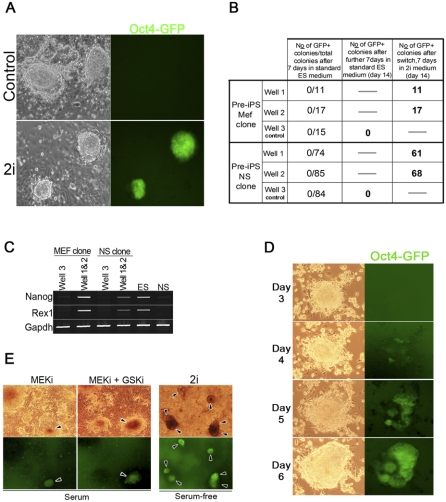
2i/LIF Induces Transition to Ground State Pluripotency (A and B) MEF and NS cell–derived pre-iPS cell clones were plated at clonal density in replica wells and cultured on a feeder layer in complete medium. After 7 d, colonies were scored and medium replenished (control) or switched to 2i. (A) Representative images of colonies 13 d after plating. Oct4 reporter (GFP) expression indicates pluripotent status. (B) Number of colonies exhibiting Oct4 reporter activation (GFP) in the indicated culture conditions. (C) RT-PCR analysis for Nanog and Rex1 in MEF and NS clones in control (well 3) and 2i conditions (wells 1 and 2). (D) Activation of Oct4GFP reporter after transfer of a pre-iPS cell colony to serum-free 2i/LIF. Duration of culture in 2i is indicated on the left. (E) Emergence of GFP-expressing colonies (arrowheads) after the addition of MEK inhibitor (i) or both MEKi and GSKi (ii) to pre-iPS cells in the presence of serum and LIF. Serum-free 2i/LIF treated cells are shown for comparison.

The isolation of 2i-iPS cells raised the question whether the inhibitors act via selection and stabilisation of cells in which full reprogramming has occurred stochastically, or by inducing the final transition to the pluripotent ground state [21]. To address this, we expanded individual colonies from infection of both MEFs and NS cells by conventional culture on a feeder layer in serum and LIF. As described above, these cells exhibit uniform ES cell–like morphology but do not express the Oct4-GFP reporter, Nanog, or Rex1. After five or more passages, cells were plated at clonal density and cultured for 7 d until macroscopic colonies were evident. Colonies were counted and duplicate wells either replenished with serum-containing medium or transferred to 2i/LIF medium. After a further 7 d activation of the Oct4 reporter, transgene was observed only in 2i/LIF (Figure 3A and 3B). All colonies from the MEF-derived clone became Oct4-GFP–positive, and 81% (129/159) from the NS cell derived clone became Oct4-GFP–positive. These populations were passaged and expanded. They express Nanog and Rex1, both of which remained absent in the cells maintained in serum (Figure 3C). The speed and high incidence of conversion without major cell death indicates an inductive process. To confirm this, we documented transition of a typical colony from Oct4-GFP–negative to –positive status over a period of 4 d in 2i culture. Figure 3D represents a visualisation of reprogramming in action. It is noteworthy that the progressive gain of Oct4-GFP throughout the continuously expanding colony is a reciprocal profile to the dynamic loss of Oct4 observed in undifferentiated inner cell masses explanted in culture without Mek inhibition[25]. In both cases, change in Oct4 is not linked to any overt morphological changes.

We also examined the effect of adding inhibitors in the presence of foetal calf serum plus LIF. The addition of 2i induced stable activation of Oct4-GFP in around 10% of emerging colonies (Figure 3E and Figure S4). GSK3 inhibitor alone had no discernible effect, but the MEK inhibitor was comparable to 2i. The reduced frequency compared with serum-free 2i culture may be because the ability of PD0325901 to suppress Erk activation is counteracted by serum factors. Nonetheless, cells expanded in serum and LIF with either MEK inhibitor only or both inhibitors acquired expression of Nanog and Rex1 (Figure S4C).

These findings indicate that factor-induced reprogramming primarily generates an undifferentiated but nonpluripotent cell state. This is a pre-pluripotent (pre-iPS) condition, however, and the final transition is efficiently induced by blockade of Erk signaling in conjunction with stimulation by LIF. GSK3 inhibition consolidates this process. We surmise that the 2i/LIF regime completes the reprogramming process by imposing a ground state upon pre-iPS cells.

### iPS Cell Generation from NS Cells Requires Few Transgene Integrations and Proceeds Efficiently without Sox2

**Figure 4:**
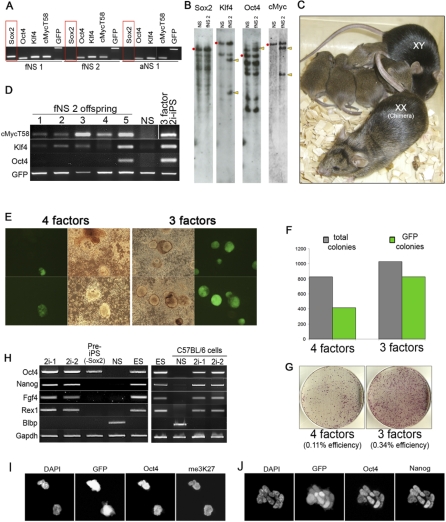
Sox2 Reduces Efficiency of NS Cell Conversion to Pluripotency (A) Genomic PCR analysis for retroviral transgenes in 2i-iPS clones generated from NS cells that were infected with the four factors. (B) Southern blot analysis for Sox2, Oct4, Klf4, and Myc retroviral integrations in fNS 2 cells. Arrowheads indicate bands corresponding to retroviral integrations and red dots those of the endogenous genes. Multiple bands in the Sox2 and Oct4 lanes are attributable to cross-hybridisation and pseudogenes, respectively. (C) Chimera generated from injection of fNS 2 2i-iPS cells into C57BL/6 host blastocyst with C57BL/6 mate and pups. Offspring coat colour demonstrates transmission of an fNS2-derived haploid genome. (D) Genomic PCR analysis for retroviral segregation in the offspring. Oct4 reporter (GFP), which is homozygous in fNS 2 cells, is used as a positive control. Absence of Oct4 and Klf4 amplification product in some offspring confirms the low transgene copy numbers. (E–G) Comparison of the reprogramming efficiency of aNS cells infected with either four factors or three factors. 8 × 10^5^ aNS cells were plated and medium switched to 2i five days after infection. (E) Examples of emerging colonies. Oct4 reporter (GFP) expression indicates pluripotent status. (F) Colony counts performed at day 8 after 2i switch. (G) Plates stained for AP 11 d after 2i switch. Puromycin selection was applied 3 d prior to staining. Reprogramming efficiency was calculated taking into account total number of GFP expressing colonies at day 11, 371, and 1137 for four- and three-factors plates, respectively, the number of plated cells, and assuming a 42% probability for a given cell being infected simultaneously with Oct4, Klf4, and c-Myc. (H) RT-PCR analysis for pluripotency markers in 2i-iPS cells (–Sox2) derived from aNS and fNS of Oct4-GFP mixed background and C57BL/6 inbred backgrounds, respectively. (I and J) Immunofluorescence staining for Oct4 and me3K27 (I) or Nanog (J) in 2i-iPS cells generated without exogenous Sox2.

Induction of the reprogramming cascade has been demonstrated to be mediated by two core factors, *Oct4* and *Sox2*, in combination with either *Klf4* and optionally *c-Myc* [1,2,26], or *Lin28* plus *Nanog* [27]. However, upon examination of 2i-iPS clones for retroviral integration by genomic PCR, we found that a *Sox2* transgene was frequently undetectable (Figure 4A). Southern hybridization analysis of one such clone confirmed absence of exogenous *Sox2*. These cells yielded functional germline chimaerism (Figure 4C), establishing that authentic 2i-iPS cells may be obtained without integration of retroviral *Sox2*. The Southern analysis indicated transgene copy numbers of one for *Oct4* and two for *Klf4* and *c-Myc* (Figure 4B). Genomic PCR revealed segregation of *Oct4* and *Klf4* transgenes amongst the germline offspring, confirming the low copy numbers (Figure 4D).

To establish that exogenous *Sox2* was indeed unnecessary, we infected adult NS cells with *Oct4*, *Klf4*, and *c-MycT58* only. As with four factors, three factor–infected NS cells rapidly and efficiently converted into pre-iPS cells that do not stably activate endogenous *Oct4* or *Nanog* when maintained in serum on feeders. However, unlike four factor–infected pre-iPS cells, which initially exhibit heterogeneity and differentiation during expansion, three factor–infected cells maintained more homogenous ES cell–like morphology (Figure S5B). Furthermore, when transferred to 2i, three factor–infected NS cells produced more colonies and activated the Oct4 reporter transgene more frequently than four factor infected–cells did (Figure 4E–4G). Three factor 2i-iPS cells were subsequently expanded and lines established from both Oct4-GFP NS cells and non-transgenic C57BL/6 NS cells. Their profile is similar to ES cells and other 2i-iPS cells in expression of *Rex1* and *Nanog* and absence of an inactive X chromosome (Figure 4H–4J). Thus, exogenous *Sox2* is not required for reprogramming NS cells. This may be because NS cells already express *Sox2* [6]. Consistent with other reports [2,26], we have not obtained iPS cells from MEFs without *Sox2* infection. Importantly, however, our data indicate that endogenous levels of Sox2 in NS cells, which are similar or slightly lower to those found in ES cells [10], are sufficient. Moreover, elevated expression of *Sox2* may destabilise pre-iPS cells and favour their differentiation.

### *Oct4* and *Klf4* Are Sufficient for NS Cell Conversion to Pluripotency

**Figure 5:**
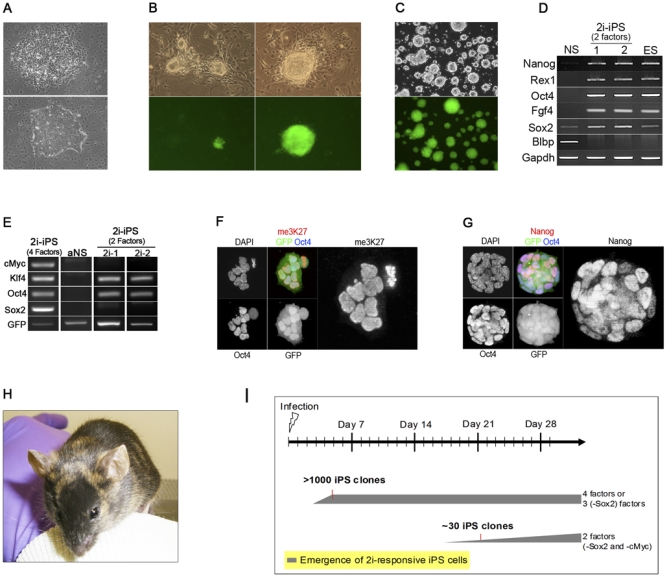
Klf4 and Oct4 Are Sufficient to Induce NS Cell Conversion to a Pre-iPS Cell State That Can Be Further Converted by 2i/LIF (A) Pre-iPS cell colonies 3 wk after infection of aNS cells with Oct4 and Klf4. (B) Appearance of clusters of cells expressing Oct4-GFP within established pre-iPS colonies, after switch into 2i/LIF. (C) Established 2i-iPS cell line generated by two-factor infection. (D) RT-PCR analysis for pluripotency markers in two-factor 2i-iPS cells. (E) Genomic PCR confirms retroviral integration of Oct4 and Klf4 only. (F and G) Immunofluorescence staining for Oct4 and me3K27 (F) or Nanog (G) in 2i-iPS cells generated without exogenous Sox2 and cMyc. (H) Adult chimera generated from injection of adult NS cell–derived two-factor 2i-iPS cells into C57BL/6 host blastocyst. (I) Schematic comparison of the reprogramming dynamics of NS cells infected with either four, three (–Sox2), or two (–Sox2 and –cMyc) factors.

Previous studies have shown that fibroblasts can be reprogrammed using *Oct4*, *Klf4*, and *Sox2*, without necessity for *c-Myc* [2,26]. We therefore tested the combination of just two factors, *Oct4* and *Klf4*. Without *c-Myc*, the rapid transition to undifferentiated morphology was not observed. However, ES cell–like colonies did appear between 2 and 3 wk post-infection. Approximately 100 colonies emerged per 8 × 10^5^ plated cells. These were more heterogenous in morphology than three factor–infected cells (Figure 5A). Nonetheless, upon transfer into 2i, around one-third of the colonies stably activated Oct4-GFP and acquired the features of 2i-iPS cells (Figure 5B–5G), including the cardinal attribute of contributing to adult chimaeras (Figure 5H).

## Discussion

We have demonstrated that NS cells can be triggered to undergo conversion to full induced pluripotency more rapidly, at higher frequency, and with fewer retroviral insertions than fibroblasts treated in parallel. Furthermore, consistent with a recent report [28], we found that NS cells can be converted without the need for exogenous *Sox2* or *c-Myc*, although omission of *c-Myc* incurs delayed kinetics and lower efficiency. Therefore the somatic cell context determines different dynamics, frequency, and transgene requirements for induced reprogramming. It is enticing to think that tissue stem cells in general might be epigenetically favoured substrates for nuclear resetting [9,10,21]. On the other hand, the presence of *Sox2* may be a specific predisposing factor in NS cells.

**Figure 6:**
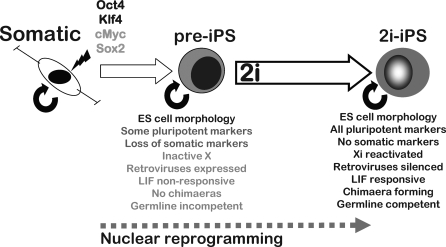
Transfection-Induced Reprogramming Commonly Stalls at a Pre-Pluripotent Stage and Can Be Advanced to Completion by Mek/Erk Inhibition Transduction of somatic cells with *Oct4*, *Klf4*, *c-Myc*, and *Sox2* transgenes gives rise at early stages to cells that have undifferentiated morphology and express some markers of ES cells. These first appearing ES cell–like cells do not convert to full pluripotency, even if continuously propagated. In the case of NS cells, partially reprogrammed cells are evident just 3 d after transduction The block to full pluripotency can be released by applying small molecule inhibitors (2i) of the Mek/Erk pathway and of glycogen synthase kinase-3 in the presence of LIF. Transition to authentic iPS cell status occurs rapidly and at high efficiency. Alternatively, 2i/LIF may be applied shortly after transduction before emergence of pre-iPS cell colonies. In this case fully reprogrammed iPS cells are isolated directly, reflecting rapid transit through, or possibly bypass of, the pre-iPS cell stage. In either scenario use of 2i enables isolation of authentic iPS cells without recourse to reporter genes or unreliable morphological assessment. In addition, reprogramming of neural stem cells to ground state pluripotency in the presence of 2i/LIF is not enhanced by exogenous *Sox2* and may proceed with only 1–2 integrations of *Oct4*, *Klf4*, and, optionally, *c-Myc*. Although, germline competent iPS cells can be obtained in standard ES cell culture conditions without use of 2i. This occurs at low frequency over 3 wk or longer. We surmise that this low-efficiency “stochastic” pathway avoids passing through or becoming trapped in the pre-iPS cell stage.

Our study has also revealed that the initial products in the reprogramming process, partially reprogrammed cells [1,2], can be promoted to full pluripotent status by applying simple, molecularly defined conditions established for derivation and propagation of ES cells [11]. Pre-iPS cells exhibit ES cell–like morphology and have down-regulated somatic cell marker genes but are characterized by the incomplete expression of pluripotency associated genes, maintained expression of retroviral transgenes, non-responsiveness to LIF, retention of epigenetic silencing of the X chromosome, and most crucially an inability to colonise chimaeras (Figure 6). They thus resemble the original Fbx15 iPS cells [2]. This pre-iPS cell state is surprisingly stable and can be propagated extensively and clonally without spontaneous transition to authentic pluripotency. However, in response to 2i plus LIF, pre-iPS cells undergo a transcriptional and epigenetic resetting that rapidly culminates in full pluripotent status with phenotypic and functional properties indistinguishable from embryo-derived ES cells. Pre-iPS cells are therefore not irrevocably misprogrammed by-products but are in fact poised on the threshold of pluripotency.

Fully reprogrammed iPS cells have previously been generated from fibroblasts [1,29] and other cell types [3,5,28] without a defined pre-iPS intermediate. iPS cells generated in this way arise at low frequency and take 3 wk or longer to emerge [14,26]. This has been attributed to requirement for an extended sequence of stochastic occurences [30]. Our finding demonstrate, however, that earlier emerging undifferentiated ES cell-like colonies, previously dismissed as incompletely or erroneously reprogrammed by-products, can be promoted rapidly and reliably to authentic pluripotency using 2i/LIF. This contrasts with a recent study that reports varying ability to further reprogramme intermediate cells using epigenetic modulators, with no evidence of chimaera formation [31].

Our data do not exclude the possibility that stable Oct4-GFP–positive fully reprogrammed cells could be generated from NS cells at low frequency and later timepoints without use of 2i [28]. Such cells might not be detected in our system because the cultures are rapidly taken over by the fast proliferating ES cell–like cells generated at the outset. These rapidly reach confluence and have to be passaged (Figure 1A). We speculate that the efficiency of initial response may be due to the relative homogeneity of our starting NS cell cultures. However, not all cells at this early stage are responsive to 2i. Further expansion is accompanied by a progressive increase in the proportion of cells that have attained the appropriate pre-iPS state to respond to 2i. This may be due both to ongoing reprogramming and to loss of misprogrammed cells to differentiation or death.

As reported by others [14,29], we do obtain some stable Oct4-GFP–expressing iPS cell colonies from infected MEFs without 2i, but only if plates are left for 3 wk or longer. These colonies appear to arise independently from the earlier emerging pre-iPS cell colonies. We surmise that this represents a delayed, low-efficiency, “stochastic” path to iPS cell generation [30]. We also noted a strong effect of genetic background on reprogramming frequency. MEFs from inbred strain 129 infected with four factors yielded many more ES cell–like colonies and subsequently 20-fold more 2i-iPS cell colonies (Figure S6) than the Oct4-GFP MEFs, which are a hybrid background between 129 and outbred MF1. It is of interest that 129 is the mouse strain from which ES cells are most readily obtained by blastocyst culture [32].

We detected very few viral integrations in fully reprogrammed cells generated from NS cells using 2i, although multiple insertions were evident in iPS cells produced from MEFs. Our data establish that 2i enables complete reprogramming of NS cells with only one or two transgene copies (Figure 4B and 4D), supporting the suggestion that a mutagenic requirement is not obligatory for reprogramming [3]. The low copy number greatly increases the feasibility of excising transgenes to generate genetically unmodified iPS cells. Furthermore, the observation that some cells acquire competence for propagation in 2i plus LIF from just 3 d after infection (Figure 2B) suggest that it might be possible to effect full reprogramming by transient infection followed by exposure to 2i/LIF.

Delineating how the nuclear reprogramming cascade unfolds is a compelling challenge in contemporary biology. The rapid, efficient, and stepwise nature of NS cell conversion with low numbers of transgene integration provides a tractable system for identifying and interrogating the underlying genetic and biochemical pathways. An immediate objective will be to elucidate how inhibiting pErk intracellular signal transduction in combination with LIF stimulation of Stat3 overcomes the block to full pluripotency, and how this effect overlaps mechanistically with previously described effects on promotion of ES cell derivation and self-renewal [11,21,33–35].

## Materials and Methods

### Cells.

Plat-E cells [36] used to produce retroviruses were maintained in DMEM containing 10% foetal calf serum (FCS), 1 μg ml^−1^ puromycin, and 10 μg ml^−1^ blasticidin S. Oct4-GFP somatic cells (NS cells and MEFs) were derived from HP165 mice [13] on a mixed, hybrid background between 129 and outbred MF1 strain background. NS cells were derived from female embryonic day 14.5 (E14.5) foetal forebrain (fNS) and female adult lateral ventricle (aNS) as described [8]. Foetal NS cells were also derived from a non-transgenic C57BL/6 male foetus. NS cells were maintained in serum-free NDiff basal RHB-A (Stem Cell Sciences, catalogue number: SCS-SF-NB-01) supplemented with 10 ng/ml of both EGF and FGF-2 [8]. MEFs were isolated from E13.5 embryos and used within three passages to avoid replicative senescence. MEFs were maintained in DMEM containing 10% FCS. ES cells and pre-iPS cells were cultured in GMEM containing 10% FCS, 1× NEAA, 1 mM sodium pyruvate, 0.1 mM 2-mercaptoethanol, supplemented with LIF (complete medium). STO fibroblasts or MEFs treated with mitomycin-C were used as feeder layers for the expansion of pre-iPS cells. 2i-iPS cells were generated and maintained without feeders in serum-free N2B27 prepared as described [37] and supplemented with LIF and 2i inhibitors [11], CHIR99021 (3μM), and PD0325901 (1 μM), obtained from the Division of Signal Transduction Therapy, University of Dundee. Chimaeras were generated by standard microinjection methodology using host blastocysts of strain C57BL/6.

### Retroviral infection and iPS cell induction.

Retroviral infection was performed as described by others [2,12] with minor modifications. Plat-E cells were seeded at 4 × 10^6^ cells per 100-mm dish. The following day, 9 μg of pMX-based retroviral vectors for expression of *Oct4*, *Sox2*, *Klf4*, or *c-MycT58* were introduced separately into Plat-E cultures using 27 μl of FuGENE 6 transfection reagent. After 24 h, the medium was replaced with 10 ml of DMEM containing 10% FCS. Target cells (MEFs and NS cells) were seeded at 8 × 10^5^ cells per 100-mm dish or 1.2 × 10^5^ cells per 35-mm dish coated with gelatin. The following day, virus-containing supernatants from Plat-E cultures were filtered through a 0.45- μm cellulose acetate filter. Equal volumes of the supernatants were mixed and supplemented with polybrene at the final concentration of 4 μg ml^−1^. Cells were incubated in the virus/polybrene-containing supernatants for 24 h, then restored to NS cell culture medium. Three days after infection, cultures were changed into complete medium containing serum and LIF. For further expansion pre-iPS cells were replated onto feeders at day 5 in complete medium and passaged every few days by trypsinisation.

Details of additional procedures and materials are provided in Text S1.

## Supporting Information

Figure S1 Nuclear Reprogramming Is Not Complete at Day 5 after NS Cell Infection (A) Adult NS cells show homogenous expression of the neural precursor marker RC2.
(B) Flow cytometry shows comparable infection and expression of control GFP retrovirus in MEFs, fNS and aNS cells.
(C and D) Immuonofluorescence for Oct4 and Nanog (C) or me3K27 (D) 5 days after infection of NS cells with the four factors. Dashed circles outline Nanog-positive cells. Arrowheads indicate the nuclear body diagnostic of the inactive X chromosome.
(506 KB PDF)

Figure S2 Generation of 2i-iPS Cells from MEFs Left panel indicates protocol procedure for the generation of 2i-iPS cells from Mef O4G cells. Right panel shows plate stained for alkaline phosphatase (AP). Indicated is also the number of 2i-iPS colonies per total number of plated cells.
(124 KB PDF)

Figure S3 2i-iPS Cells Exhibit Properties of ES Cells (A) Northern analysis for Oct4 expression in ES, pre-iPS, pre-iPS GFP sorted, and two independent 2i-iPS clones.
(B) RNA FISH for Tsix in XX 2i-iPS and XX pre-iPS cells. Arrows indicate Tsix RNA nascent transcripts. Percentages of cells exhibiting Tsix allelic expression are indicated.
(C) Differentiation (left) and self-renewal (right) of 2i-iPS cells in medium with serum and without or with LIF respectively. Oct4 reporter (GFP) expression indicates pluripotent status.
(D) 2i-iPS cells remain undifferentiated in serum free media supplemented with LIF and Bmp4.
(448 KB PDF)

Figure S4 Inhibition of MEK Releases pre-iPS Cells from Reprogramming Block (A) Number of colonies expressing Oct4 reporter (GFP) derived from passage 1 pre-iPS cells replated at 1 × 10^5^/well in the indicated culture conditions. Control conditions refer to ES medium and feeders alone, while tested conditions refer to the addition of MEK inhibitor (i), GSKi, or both MEKi and GSKi. Puromicin selection for Oct4 reporter expression was applied to control and GSKi cultures when these reached confluency.
(B) Cultures of Oct4 reporter (GFP)–expressing cells generated in the presence of MEKi (left) or MEKi and GSKi (right).
(C) Reverse-transcription PCR (RT-PCR) analysis for the pluripotency markers Nanog and Rex1 in the indicated cultures.
(630 KB PDF)

Figure S5 Sox2 Expression Is Not Necessary for 2i-iPS Cell Generation (A) RT-PCR analyses for Oct4, Sox2, Klf4, and Klf2 in ES, NS and MEFs.
(B) Cell morphology of intermediate state iPS cells infected with four factors (top panel) and three factors (–Sox2). Dashed line highlights clusters of cells with round and retractile morphology.
(C) RT-PCR analysis of total Sox2 and Oct4 levels in fNS cells infected with four factors. Analyses were performed at day (d) 0, 3, 5, passage 10 (P10), and on 2i-iPS cells established from P10 cells.
(396 KB PDF)

Figure S6 Mouse Genetic Background Affects Reprogramming Efficiency (A) Immunostaning for Nanog of 129 MEFs infected with four factors and dsRed (RFP).The cells have been isolated using serum and LIF culture conditions only. Absence of RFP indicates retroviral silencing.
(B) Left panel indicates protocol procedure for the generation of 2i-iPS cells from 129 MEFs. Right panel shows plate stained for alkaline phosphatase (AP). This represents a parallel infection with Mef O4G plate in Figure S3. Indicated is also the reprogramming efficiency that was calculated taking into account total number of AP-positive colonies, approximately 900, the number of plated cells, 8 × 10^5^, and assuming a 66% probability for a given cell being infected simultaneously with *Oct4*, *Klf4*, *Sox2*, and *c-Myc*.
(437 KB PDF)

Text S1 Details for PCR, Northern Blot, Southern Blot, Immunostaining, and RNA FISH Procedures The text also provides list of primer sequences, antibodies, and plasmids used.
(92 KB DOC)

## Abbreviations

2i: dual inhibition (PD0325901 plus CHIR99021)
2i-iPS cell: induced pluripotent stem cell obtained after exposure to 2i conditions
aNS: adult brain derived neural stem cell line
Erk: extracellular signal regulated kinases 1 and 2
ES cell: embryonic stem cell
FCS: foetal calf serum
fNS: foetal forebrain derived neural stem cell line
GFP: enhanced green fluorescence protein
GSK3: glycogen synthase kinase 3
H3K27: histone H3 amino acid lysine 27
iPS cell: induced pluripotent stem cell
LIF: leukaemia inhibitory factor
Me3H3K27: histone H3 with trimethylated amino acid lysine 27
MEF: mouse embryo fibroblast
Mek: mitogen activated protein kinase/ERK kinase
NS cell: neural stem cell propagated in adherent culture without differentiation
pre-iPS cell: undifferentiated but incompletely reprogrammed cell responsive to 2i
RT-PCR: reverse-transcription PCR

## Note Added in Proof

To date, we have observed no tumours in iPS chimaeras or germline progeny up to 6 mo.
