# Supplementary material for: Promotion of Reprogramming to Ground State Pluripotency by Signal Inhibition
Source: PLoS Biol. 2008 Oct 21;6(10):e253. doi: 10.1371/journal.pbio.0060253 (PMC2570424; doi:10.1371/journal.pbio.0060253)
Supplement: Supplementary file 1 [file pbio.0060253.sg001.pdf]

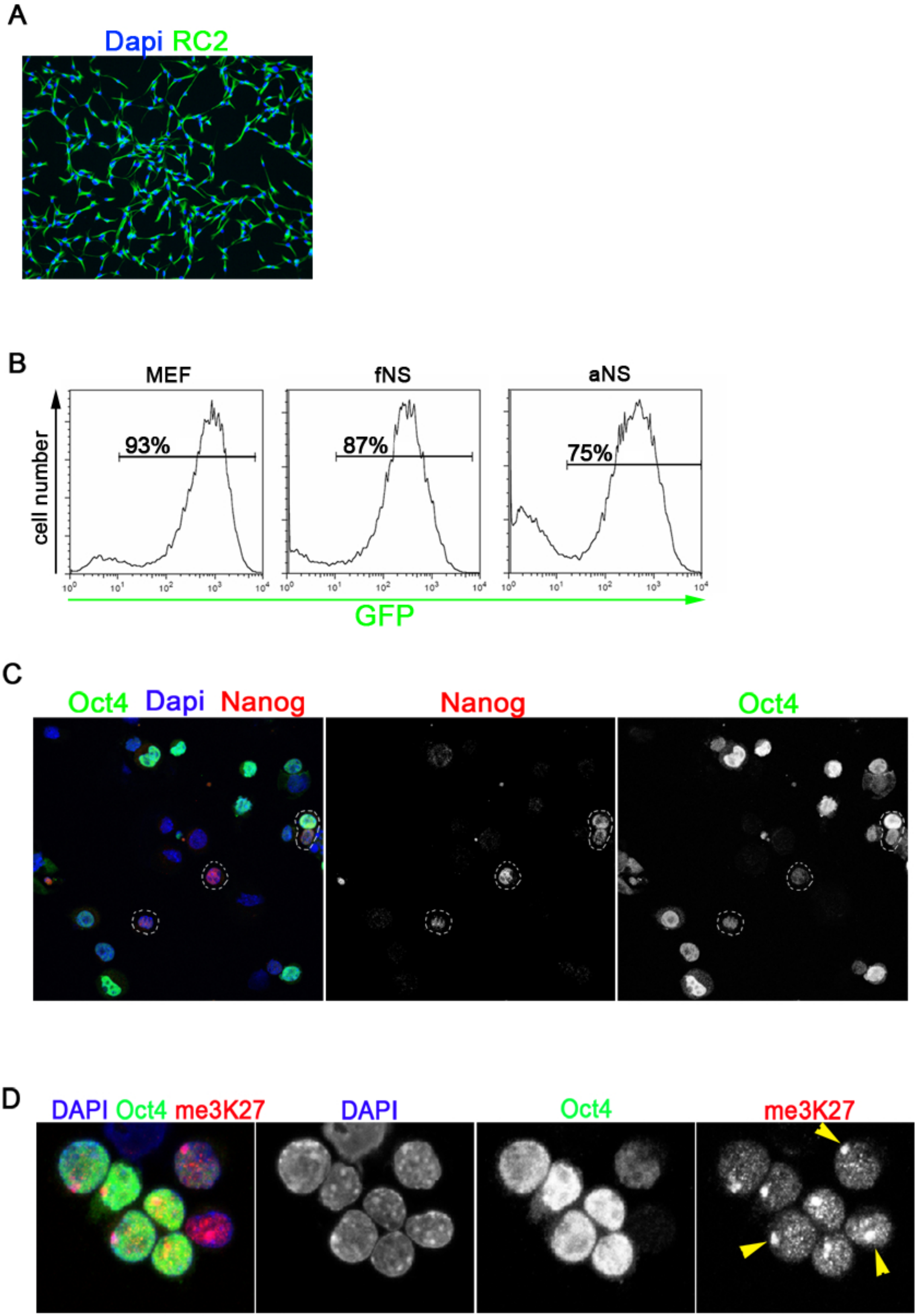
A
Dapi RC2
B
MEF
fNS
aNS
cell number
93%
87%
75%
GFP
C
Oct4 Dapi Nanog
Nanog
Oct4
D
DAPI Oct4 me3K27
DAPI
Oct4
me3K27
