# Supplementary material for: Promotion of Reprogramming to Ground State Pluripotency by Signal Inhibition
Source: PLoS Biol. 2008 Oct 21;6(10):e253. doi: 10.1371/journal.pbio.0060253 (PMC2570424; doi:10.1371/journal.pbio.0060253)
Supplement: Supplementary file 2 [file pbio.0060253.sg002.pdf]

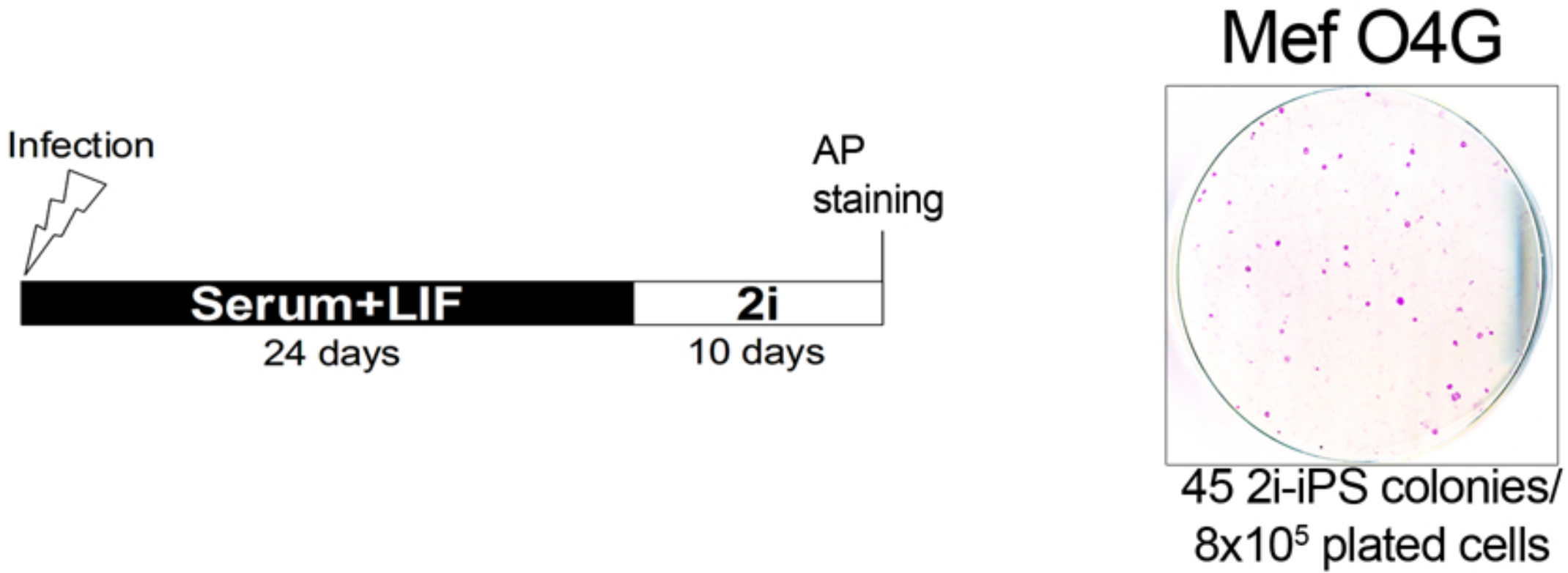

Infection
AP
staining
Serum+LIF
2i
24 days
10 days
Mef O4G
45 2i-iPS colonies/
8x10^5 plated cells
