# Supplementary material for: Promotion of Reprogramming to Ground State Pluripotency by Signal Inhibition
Source: PLoS Biol. 2008 Oct 21;6(10):e253. doi: 10.1371/journal.pbio.0060253 (PMC2570424; doi:10.1371/journal.pbio.0060253)
Supplement: Supplementary file 3 [file pbio.0060253.sg003.pdf]

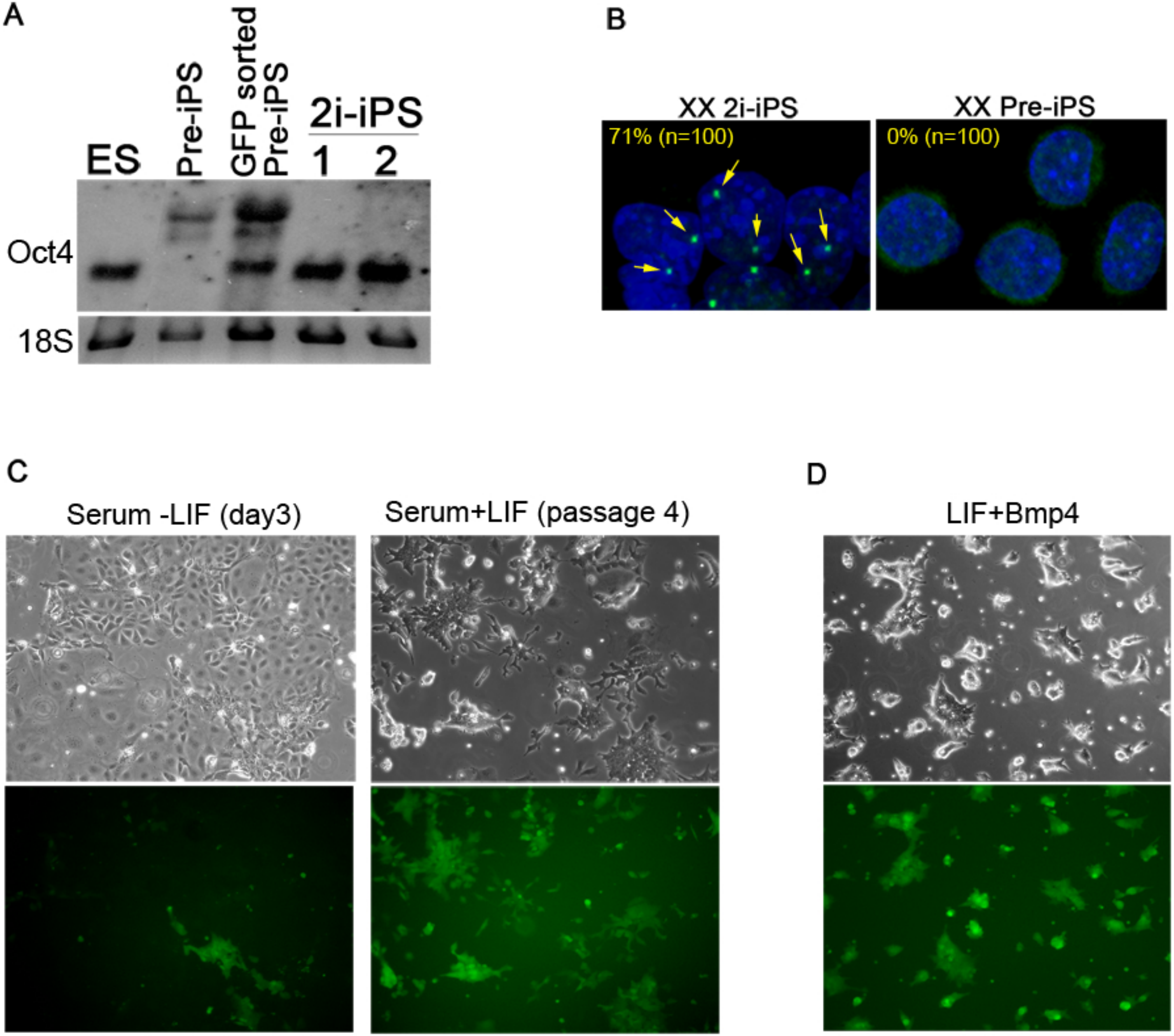
A
ES
Pre-iPS
GFP sorted Pre-iPS
2i-iPS
1
2
Oct4
18S
B
XX 2i-iPS
71% (n=100)
XX Pre-iPS
0% (n=100)
C
Serum -LIF (day3)
Serum+LIF (passage 4)
D
LIF+Bmp4
