# Supplementary material for: Promotion of Reprogramming to Ground State Pluripotency by Signal Inhibition
Source: PLoS Biol. 2008 Oct 21;6(10):e253. doi: 10.1371/journal.pbio.0060253 (PMC2570424; doi:10.1371/journal.pbio.0060253)

A

Culture conditions	control	GSKi	MEKi (2μM)	MEKi (3μM)	MEKi (3μM) GSKi
No of GFP expressing colonies	**0**	**0**	**51**	**55**	**40**

B

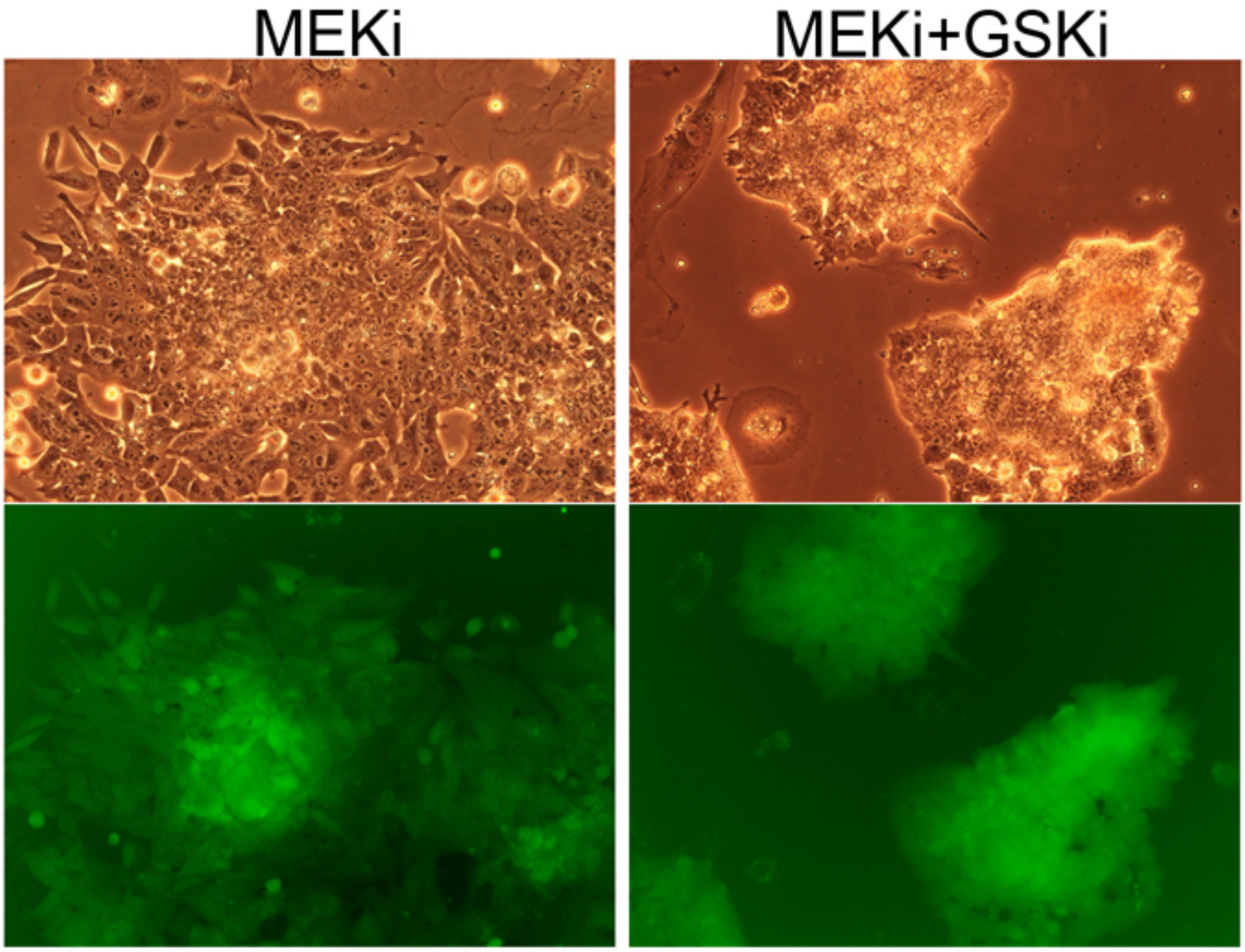

C

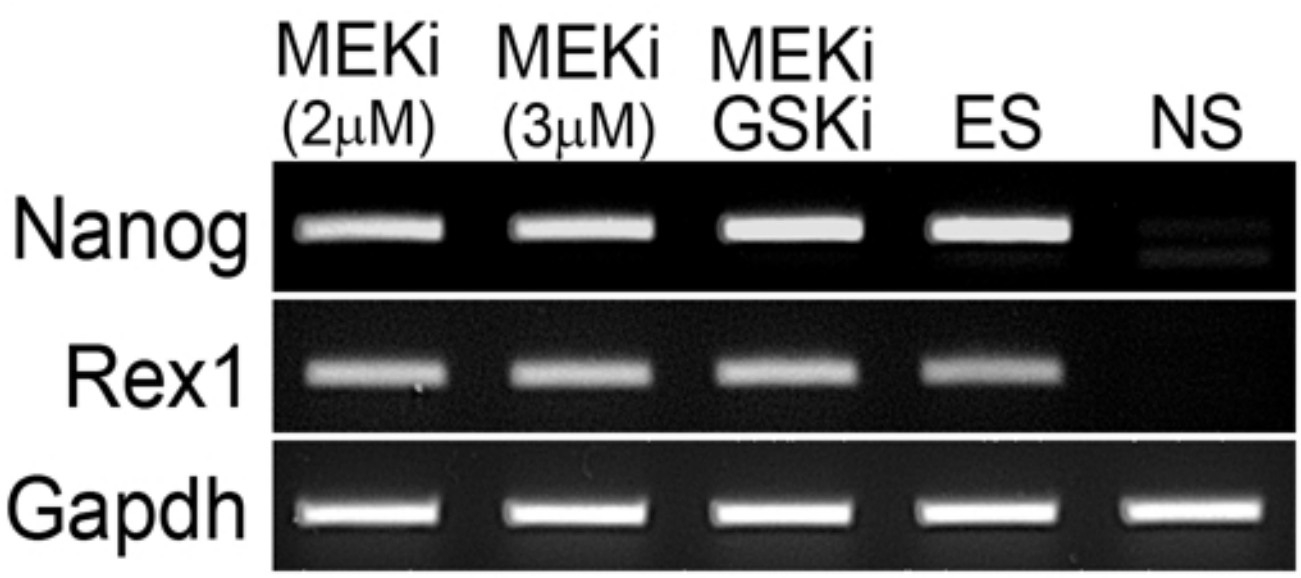

Supplement: Supplementary file 4 [file pbio.0060253.sg004.pdf]
