# Supplementary material for: Promotion of Reprogramming to Ground State Pluripotency by Signal Inhibition
Source: PLoS Biol. 2008 Oct 21;6(10):e253. doi: 10.1371/journal.pbio.0060253 (PMC2570424; doi:10.1371/journal.pbio.0060253)
Supplement: Supplementary file 5 [file pbio.0060253.sg005.pdf]

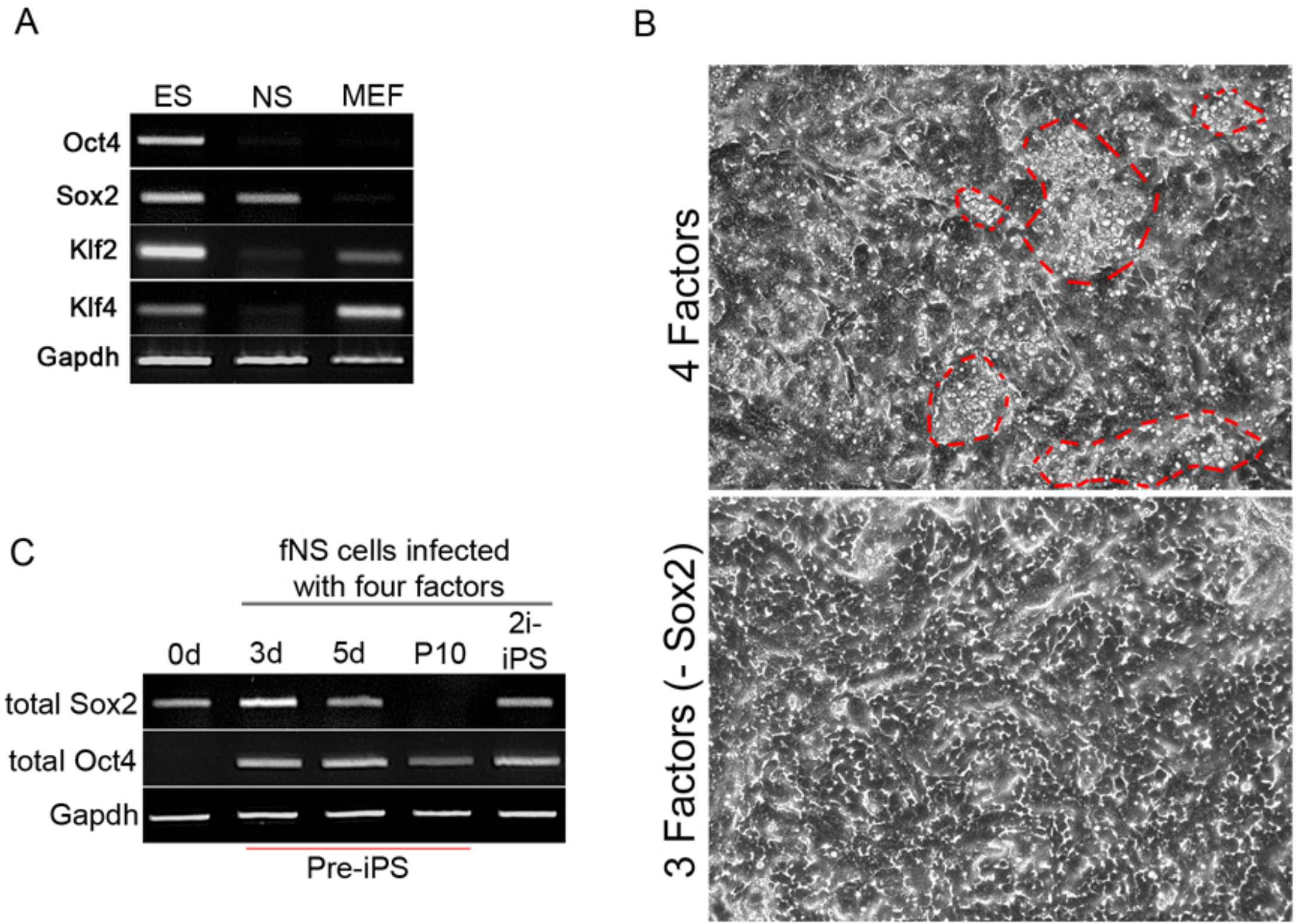
A
ES
NS
MEF
Oct4
Sox2
Klf2
Klf4
Gapdh
B
4 Factors
3 Factors (- Sox2)
C
fNS cells infected
with four factors
0d
3d
5d
P10
2i-
iPS
total Sox2
total Oct4
Gapdh
Pre-iPS
