# Supplementary material for: Promotion of Reprogramming to Ground State Pluripotency by Signal Inhibition
Source: PLoS Biol. 2008 Oct 21;6(10):e253. doi: 10.1371/journal.pbio.0060253 (PMC2570424; doi:10.1371/journal.pbio.0060253)
Supplement: Supplementary file 6 [file pbio.0060253.sg006.pdf]

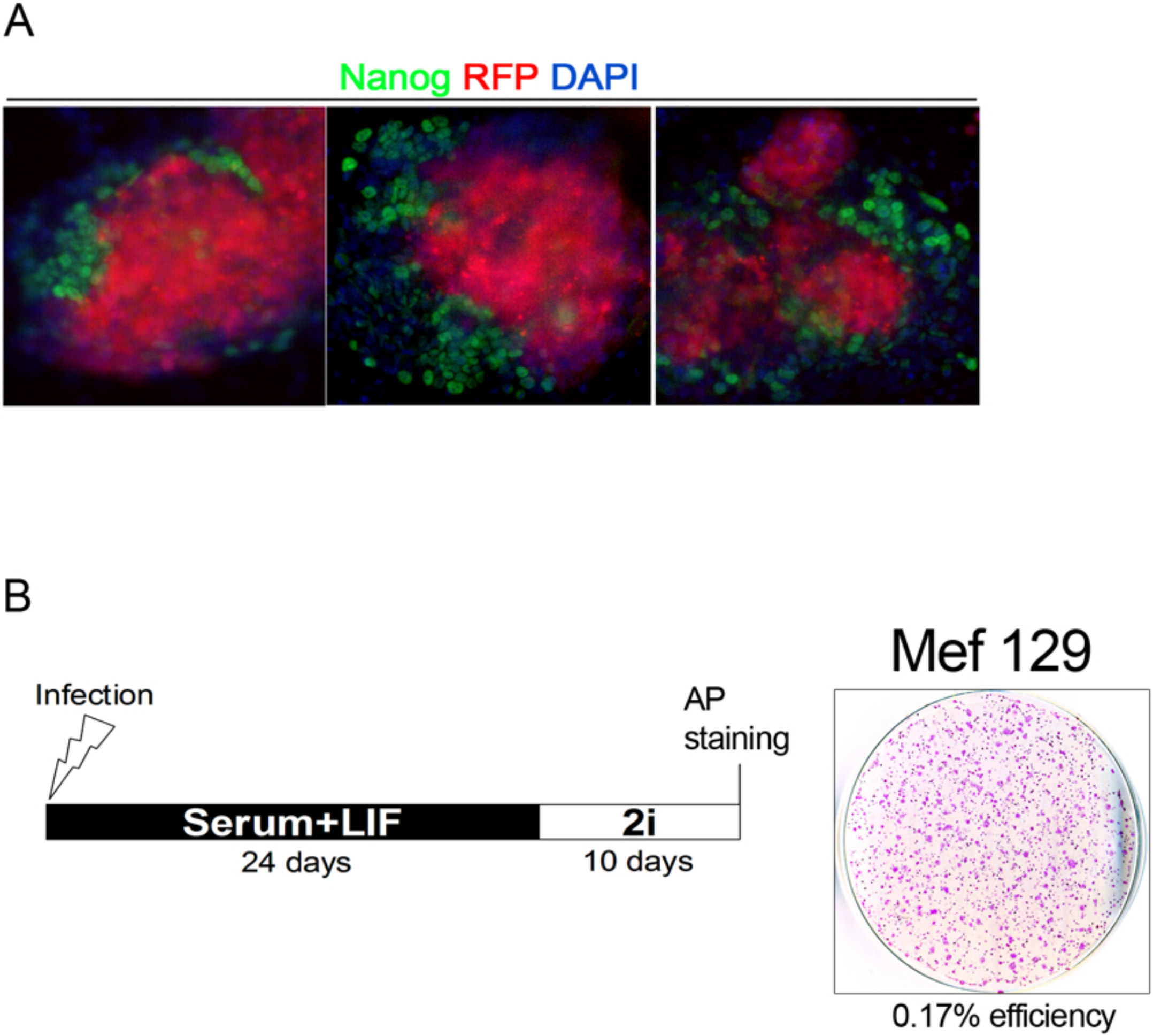
A
Nanog RFP DAPI
B
Infection
AP
staining
Serum+LIF
2i
24 days
10 days
Mef 129
0.17% efficiency
