# Supplementary material for: Promotion of Reprogramming to Ground State Pluripotency by Signal Inhibition
Source: PLoS Biol. 2008 Oct 21;6(10):e253. doi: 10.1371/journal.pbio.0060253 (PMC2570424; doi:10.1371/journal.pbio.0060253)
Supplement: Supplementary file 7 [file pbio.0060253.sd001.doc]

**Text S1**

#### Plasmids pMXs-gw plasmids; pMXs-Oct4, pMXs-Klf4, pMXs-cMycT58 and pMXs-Sox2 were obtained from Addgene repository. pMXs-GFP plasmid was a gift from Professor Toshio Kitamura.

#### RT-PCR for Marker Genes Total RNA was extracted using RNeasy kit (Qiagen), and cDNA generated using superscript III (Invitrogen). PCR was performed using Taq Polymersase (Qiagen) or Phusion™ High-Fidelity DNA Polymerase (New England BioLabs). Primer sequences and cycling conditions are described in primer table (see below).

**Antibodies** The following antibodies and dilutions were used: Oct4 (1:100) from Santa Cruz Biotechnology (C-10, cat no: sc-5279), trimethyl H3-K27 (1:500) was a gift from Thomas Jenuwein, Nanog (1:200) from abcam (cat no: ab21603-100) and RC2 (1:50) mouse IgM from DSHB. Immunofluorescence stainings were performed as previously described[1,2,3].

**RNA FISH** was carried out as previously described[4]. The Tsix probe (pTsix5’) containing Tsix promoter and 5’ region was a gift from Neil Brockdorff.

**Analysis of genomic integration** Genomic PCR for retroviral transgenes was carried out using the “retro” primers listed in the Table below. Southern hybridization was performed using the GE Healthcare AlkPhos Direct Labeling and Detection System with CDP-Star according to the manufacturer’s specifications. Genomic DNA was digested with EcoRI and transfer blots hybridized individually with full length Sox2, Klf4 or cMyc cDNA probes.

**Northern hybridization** was performed using the GE Healthcare AlkPhos Direct Labeling and Detection System with CDP-Star according to the manufacturer’s specifications. Total RNA was hybridized using full length Oct4, including UTR sequence.

**Imaging and flow cytometry** Slides were analyzed on a confocal microscope (Leica TCS SP5) and processed with Leica software and Adobe Photoshop. Images of live cells were captured with a Leica CTR microscope and processed with Leica software and Adobe Photoshop. Flow cytometry analyses were performed using a Dako Cytomation CyAn ADP high-performance cytometer with FlowJo and Summit software. Cell sorting was performed using a MoFlo high-speed cell sorter.

**Quantitative RT-PCR analysis:** Relative expression levels of Fgf4, Nanog, Nr0b1 and Rex1 were determined using the TaqMan Fast Universal PCR Master Mix (Applied Biosystems) and the FAM-labeled TaqMan probes, Mm00438917_m1, Mm02384862_g1, Mm00431729_m1 and Mm03053975_g1 respectively. Average threshold cycles were determined from triplicate reactions on a 7900 HT Fast Real-Time PCR System (Applied Biosystems), and the levels of gene expression were normalized to β-actin (VIC-labeled TaqMan probe, 4352341E). Relative expression levels of Blbp and Olig2 were determined using gene-specific primers and FAM-labeled probes (no. 58 and 21 respectively) from the Universal Probe Library (UPL) Set (Roche). The primers for Blbp were: 5’- aaccagcatagatgacagaaactg -3’ (forward) and 5’- acttctgcacatgaatgagctt -3’ (reverse), and the primers for Olig2 were: 5’- agaccgagccaacaccag-3’ (forward) and 5’- aagctctcgaatgatccttcttt-3’ (reverse). Average threshold cycles for Blbp and Olig2 were determined from triplicate reactions, and the levels of gene expression were normalized to GAPDH (VIC-labeled TaqMan probe, 4352339E).

**Primer table**

**Text S1 references**

1. Silva J, Mak W, Zvetkova I, Appanah R, Nesterova TB, et al. (2003) Establishment of histone h3 methylation on the inactive X chromosome requires transient recruitment of Eed-Enx1 polycomb group complexes. Dev Cell 4: 481-495.

2. Silva J, Chambers I, Pollard S, Smith A (2006) Nanog promotes transfer of pluripotency after cell fusion. Nature 441: 997-1001.

3. Conti L, Pollard SM, Gorba T, Reitano E, Toselli M, et al. (2005) Niche-independent symmetrical self-renewal of a mammalian tissue stem cell. PLoS Biol 3: e283.

4. Heard E, Rougeulle C, Arnaud D, Avner P, Allis CD, et al. (2001) Methylation of histone H3 at Lys-9 is an early mark on the X chromosome during X inactivation. Cell 107: 727-738.
